# Risks and benefits of oral immunotherapy for IgE-mediated cow's milk allergy

**DOI:** 10.1186/1939-4551-8-S1-A204

**Published:** 2015-04-08

**Authors:** Wilson Rocha Filho, Fernanda Junqueira Flausino, Rosiléa Alves Silva, Fabiana Maria Silva, Jorge Andrade Pinto, Lorena Nunes

**Affiliations:** 1 Fhemig, Brazil; 2 Hospital João Paulo II, Brazil; 3 Hospital Infantil João Paulo II – Fhemg, Brazil; 4 Federal University of Minas Gerais, Brazil

## Background

We sought to evaluate whether oral immunotherapy (OIT) is safe and efficacious in desensitizing children with cow's milk allergy (CMA).

## Methods

We study 23 children with CMA that met the following criteria: clinical history of IgE-mediated CMA in the last 6 months and specific IgE (sIgE) for cow's milk (CM) ≥ 15 kU/L (> 2 years) or ≥ 5 kU/L (< 2 years). On study entry, basal serum levels for sIgE for CM and casein plus sIgG4 for casein were obtained. The study was divided into 2 stages. In the first 12 months, patients were randomly assigned in a double-blind manner to placebo or active OIT. At the end of the first 12 months period, patients submitted to milk double-blind placebo-controlled food challenge (DBPCFC). The study was unblinded in this time and patients on the placebo group started on a 24 months active OIT period. Children on active OIT completed 12 more months of treatment. sIgE for CM and casein and sIgG4 for casein were obtained throughout the study.

## Results

15 children completed treatment: 11 in the active group and 4 in the control. The median frequency for total reactions in active group was 56,6% versus 19,4% in the placebo group (p < 0,05). At the end of the first 12 months period, the test group reacted with an average of 10 g of whole milk in DBPCFC versus 4.1 g in the placebo group (p > 0.05). After 12 months active OIT period, 4 patients had a negative challenge, and after 24 months 3 more patients had a negative challenge. Among the 6 participants who reacted, there was an increase an average of 5,2 g dose threshold to cause reactions to milk during the period between 12 and 24 months of treatment. There was an increase in the frequency of symptoms to the average dose of 1,9 g, with subsequent decrease. In cases who tolerated 12 months, there was a significant increase of sIgG4 for casein at 12 months from baseline, compared to non-tolerant. The evolution of serum levels over time, by adjusting the graphs of smoothing, showed a reduction of sIgE for CM and casein at 24 months and an increase of sIgG4 for casein in the first 12 months.

## Conclusions

Milk OIT seems to be effective in the induction of clinical desensitization and immunomodulation in children with CMA, but the risk-benefits should be weighed.

